# Variability of clinical presentation and diagnostic challenges in osteoarticular sporotrichosis: a case series

**DOI:** 10.1590/S1678-9946202668003

**Published:** 2026-01-30

**Authors:** Renê Donizeti Ribeiro de Oliveira, Roberto Martinez, Gilberto Gambero Gaspar, Paulo Louzada, Rodrigo de Carvalho Santana

**Affiliations:** 1 Universidade de São Paulo Faculdade de Medicina de Ribeirão Preto Departamento de Clínica Médica São Paulo Brazil Universidade de São Paulo, Faculdade de Medicina de Ribeirão Preto, Departamento de Clínica Médica, Ribeirão Preto, São Paulo, Brazil; 2 Universidade de São Paulo Faculdade de Medicina de Ribeirão Preto Departamento de Medicina Social São Paulo Brazil Universidade de São Paulo, Faculdade de Medicina de Ribeirão Preto, Departamento de Medicina Social, Ribeirão Preto, São Paulo, Brazil

**Keywords:** Sporotrichosis, Osteoarticular sporotrichosis, *Sporothrix* spp, Fungal osteomyelitis

## Abstract

Osteoarticular sporotrichosis is the most common extracutaneous type of the disease and may occur either concomitantly with cutaneous lesions or as isolated musculoskeletal disease, the latter frequently resulting in delayed diagnosis. We describe five confirmed cases of osteoarticular sporotrichosis diagnosed between 2002 and 2023 at a university hospital in Brazil. Diagnosis was confirmed by fungal culture, with serology and histopathology used as complementary methods. Clinical and epidemiological data, radiologic findings, treatment, and outcomes were analyzed. Patients were 39 to 67 years of age and all had chronic conditions or alcoholism. Joint involvement most frequently affected the knee (four cases), followed by the wrist (three cases), and small joint of the hands (two cases), often with bone and periarticular tissue involvement. Three patients had concomitant cutaneous involvement. Diagnostic delays were frequent, reflecting the nonspecific clinical presentation and the tendency to misattribute symptoms to other musculoskeletal conditions. All patients received antifungal therapy with itraconazole and/or amphotericin B. Relapses were recorded in two patients, and there were irreversible sequelae such as chronic arthritis, joint stiffness, or deformity in all cases. Osteoarticular sporotrichosis should be considered in the differential diagnosis of chronic musculoskeletal conditions, including arthritis, osteomyelitis, synovitis, bursitis, and tenosynovitis, particularly in endemic regions. Early recognition and prolonged antifungal therapy are essential to achieve cure and prevent complications.

## INTRODUCTION

Infections caused by *Sporothrix* spp. most frequently involve the skin, subcutaneous tissue, and regional lymphatic chains. Less commonly, sporotrichosis may disseminate, affecting bones, joints, lungs, the central nervous system, and other organs^1^. Sporotrichosis has five clinical types: cutaneous, immunoreactive, mucosal, osteoarticular, and systemic^2^. The classical route of transmission is sapronosis, which results from traumatic inoculation of fungal propagules from contaminated plant material or soil into the skin. This type is commonly associated with occupational or environmental exposure among gardeners, farmers, florists, and rural workers. Recently, zoonotic transmission has gained increasing importance, particularly via infected domestic cats, in which infection occurs via scratches, bites, or contact with exudates from infected animals and may cause outbreaks of human sporotrichosis^2^.

Osteoarticular involvement is the second most common manifestation after the cutaneous type of sporotrichosis, and is classified as primary (unifocal/localized) or multifocal (systemic) according to the route of infection. In the primary type, the fungus spreads from a cutaneous lesion or by direct inoculation into bone or joint tissues. In the multifocal type, dissemination occurs hematogenously, affecting multiple osteoarticular sites depending on the immune status of the host^3^. Historical reviews estimate the prevalence of bone and joint types as low, as 0.03%–0.4%^4^. In Brazil, a review of cases reported between 1907 and 2020 found osteoarticular involvement in approximately 1% of patients with defined clinical forms^5^. A recent Sao Paulo cohort identified only one osteoarticular disease case among 260 sporotrichosis patients (0.38%)^6^. Beyond Brazil, osteoarticular types have been reported in several other countries as USA, Germany, Colombia, Peru, Japan, Thailand, Mexico, India, Malaysia, and South Africa^3^.

Some patients with osteoarticular sporotrichosis lack cutaneous lesions, which may contribute to delayed diagnosis, as the symptoms often mimics that of other musculoskeletal disorders. This case series describes five patients with osteoarticular sporotrichosis, emphasizing the variability in presentation, diagnostic challenges, and therapeutic management.

### Ethics

The study complied with Resolution Nº 466/2012 of the Brazilian National Health Council (CNS), which establishes ethical principles for research involving human subjects. Approval was obtained from the Institutional Ethics Committee for the report of all six cases (CAAA Nº 68103123.0.0000.5440). Written informed consent was waived due to the retrospective nature of the study, there was a difficulty in contacting most patients who were no longer under follow-up, which was based on the commitment and responsibility of the principal investigator to ensure anonymization and protection of all personal data.

## CASE REPORTS

All five patients—four men and one woman, aged 39–67 years—lived in Sao Paulo and Minas Gerais states, Brazil. They were diagnosed with osteoarticular sporotrichosis between 2002 and 2023 and treated at the University Hospital of the Ribeirao Preto Medical School, University of Sao Paulo. Radiologic studies and routine laboratory tests were performed to investigate joint involvement. In all cases, diagnosis was confirmed by isolation of *Sporothrix* spp. in culture; serology (counterimmunoelectrophoresis) and histopathology were used as complementary methods. The detailed clinical descriptions of these cases are provided below. Tables 1 and 2 summarize laboratory and imaging findings at the time of diagnosis.

**Table 1:** Clinical and laboratory characteristics of osteoarticular sporotrichosis cases

Case	1	2	3	4	5
Age	51	62	58	67	39
Sex	Male	Male	Male	Male	Female
Clinical presentation	Unifocal osteoarticular	Lymphocutaneous and unifocal osteoarticular	Multifocal* osteoarticular	Cutaneous and unifocal osteoarticular	Cutaneous and unifocal osteoarticular
Histopathology findings	Not done	Synovial tissue biopsy: lymphohistiocytic infiltrate rich in plasma cells.	Juxta-articular skin biopsy: Lymphohistiocytic infiltrate, neutrophils, epithelioid granulomas, and Sporothrix spp. yeasts.	Femoral condyle biopsy: fibrotic stroma with areas of lymphocytic infiltrate.	Synovial tissue biopsy: synovium and adjacent adipose tissue with chronic fibrosing inflammatory process
Fungal culture	*Sporothrix* spp. isolated from SF and nodule aspirate	*Sporothrix* spp. isolated from SF	*Sporothrix* spp. isolated from SF	*Sporothrix* spp. isolated from the femoral condyle (biopsy) and pus from a popliteal abscess.	*Sporothrix* spp. isolated from bone and synovial tissue of the left knee (biopsy).
Anti-*Sporothrix* spp. antibodies (CIEP) **	1:32	1:128	1:256	1:32	1:64
SF cytology	leukocytes: 16,000/µL (N-73%, Ly-21%, M-6%)	leukocytes: 14,100/µL (N-80%, Ly-19%, M-1%)	leukocytes: 6,600/µL (N-82%, Ly-17%, M-1%)	Not done	Not done

* presumptive;

** performed at the time of diagnosis; CIEP = counterimmunoelectrophoresis; Ly = lymphocyte; M = monocyte; N = neutrophil; SF = synovial fluid.

**Table 2:** Radiological features, antifungal therapy, and outcomes in patients with osteoarticular sporotrichosis

Case	Osteoarticular presentation	Radiological findings at diagnosis	Sequential antifungal therapy	Outcome
1	Unifocal (Arthritis and osteomyelitis)	Radiography of the left hand: periarticular osteopenia, bone lysis in the scaphoid and radial styloid, bone sclerosis, and decreased joint space in the wrist.	AmB deoxycholate: cumulative dose of 200 mg; oral itraconazole: 30 months.	Cured with sequelae – regression of inflammation, residual pain, mild motion limitation, and synovial thickening. No other complications.
2	Unifocal (Arthritis and tenosynovitis)	NMR of L knee: Diffuse synovial thickening, small marginal bone erosions, inflammatory cyst next to the popliteal tendon, and moderate joint effusion	Saturated solution of potassium iodide: four months; oral itraconazole: 24 moths; liposomal AmB: 50 mg/day, 46 infusions over six weeks; oral itraconazole: 24 moths; intra-articular AmB deoxycholate; oral itraconazole: 12 moths.	Cured with sequelae – arthritis remitted; residual synovial thickening and occasional pain due to secondary osteoarthritis. Patient relapsed during follow-up.
3	Multifocal* (Arthritis and tenosynovitis)	NMR of R hand: Synovial thickening and contrast enhancement in the tendon sheath of the extensor muscles, in some of the flexor tendons, and the second metacarpal phalangeal joint. US of lower limbs: synovial thickening in the R fibula tendons and L extensor tendons; nodulations in the R thigh and quadriceps subcutaneous tissue.	Oral itraconazole: 12 months; terbinafine: two months; liposomal AmB: 50 mg/day, ∼98 infusions over 14 weeks; oral itraconazole: 14 months; oral itraconazole: ∼ 45 months.	Cured with sequelae – partial control after multiple relapses; residual left knee swelling and right wrist deformity with limited range of motion. Patient relapsed during follow-up. No other complications.
4	Unifocal (Arthritis)	NMR of the R knee: Erosion of articular surfaces, subchondral cysts on the femoral condyles, tibial plateaus, and patella; small joint effusion; reduction of the medial and lateral menisci; abscess in the popliteal fossa measuring approximately 15×8.5cm.	Itraconazole: 30 months	Cured with sequelae – partial improvement, residual knee enlargement and stiffness. No other complications.
5	Unifocal (Arthritis and osteomyelitis)	NMR of the L knee: Intense irregularity of the joint surfaces, with cartilage erosion and areas of subchondral bone exposure. Moderate joint effusion with signs of synovial thickening. Bulky collection that maintains contact with the joint space next to the external femoral condyle.	Itraconazole: 27 months	Cured with sequelae – skin healing and reduced swelling; persistent ankylosis and functional loss of the knee. No other complications

* presumptive; AmB = amphotericin B; L = left; R = right; NMR = nuclear magnetic resonance; US = ultrasonography.

### Case 1

A 51-year-old former rural worker with diabetic neuropathy and prior exposure to soil and organic material presented chronic arthritis of the left wrist, with edema, nodules, reduced mobility, and four cysts up to 3 cm in diameter. No cutaneous lesions were documented at presentation, nor reported in the past. On examination, he showed stiffness of the metacarpophalangeal joints and hypotrophy of the thenar, hypothenar, and dorsal muscles of the left hand. *Sporothrix* spp. was isolated from wrist synovial fluid aspiration, confirming unifocal osteoarticular sporotrichosis. HIV serology was negative. He was treated with amphotericin B deoxycholate (cumulative dose: 200 mg), followed by itraconazole (200 mg/day) for 30 months. Inflammation gradually subsided, leaving mild residual pain, limited range of motion, and a persistent wrist nodule. Anti-*Sporothrix* antibodies declined from 1:31 at diagnosis to 1:4 at final evaluation.

### Case 2

A 62-year-old rural worker with chronic arterial disease and chronic obstructive pulmonary disease, on inhaled corticosteroids, developed lymphocutaneous sporotrichosis following a puncture wound on the right hand from a plant fragment. HIV serology was negative. Lesions regressed partially after four months of saturated solution of potassium iodide therapy, but the patient discontinued treatment. Three months later, he developed arthritis with effusion on the left knee, and *Sporothrix* spp. was isolated from knee synovial fluid aspiration, confirming unifocal osteoarticular sporotrichosis. Itraconazole (400 mg/day) led to partial improvement, but relapses occurred despite prolonged therapy, with repeated positive cultures. He was then treated with liposomal amphotericin B (50 mg/day; 15 doses over 54 days), followed by oral itraconazole, with only partial response. Subsequently, intra-articular amphotericin B deoxycholate was administered in combination with oral itraconazole for 12 months. Arthritis ultimately regressed, though some residual joint enlargement and pain persisted (secondary osteoarthritis). Anti-*Sporothrix* antibodies declined from 1:128 at presentation to 1:4 at final evaluation.

### Case 3

A 58-year-old man with frequent exposure to soil and plants in rural settings and comorbidities including type 2 diabetes, arterial hypertension, smoking, and prior coronary surgery initially presented a painful erythematous macule on the left leg, which was compatible with erythema nodosum, accompanied by fever and arthralgia. No other cutaneous lesions were observed or reported, and HIV serology was negative. Over six months, arthritis progressively involved the wrists, metacarpophalangeal and proximal interphalangeal joints of both hands, and the left knee. *Sporothrix* spp. was isolated from knee synovial fluid, confirming infectious arthritis. Additional joint involvement was inferred from clinical and radiological findings, and the case was classified as presumptive multifocal osteoarticular sporotrichosis. He was treated with itraconazole (400 mg/day) for 12 months with partial improvement, but symptoms worsened after switching to terbinafine. Subsequent therapy with liposomal amphotericin B (50 mg/day, ∼98 infusions over 14 weeks) followed by itraconazole improved his condition, although early discontinuation led to multiple relapses over the following decade, including synovial abscess formation. After prolonged itraconazole therapy (up to 45 months), the patient achieved cure with sequelae, and had residual knee swelling and wrist deformity with limited motion. Anti-*Sporothrix* antibodies declined from 1:256 at diagnosis to 1:32 at final evaluation.

### Case 4

**Figure 1:**
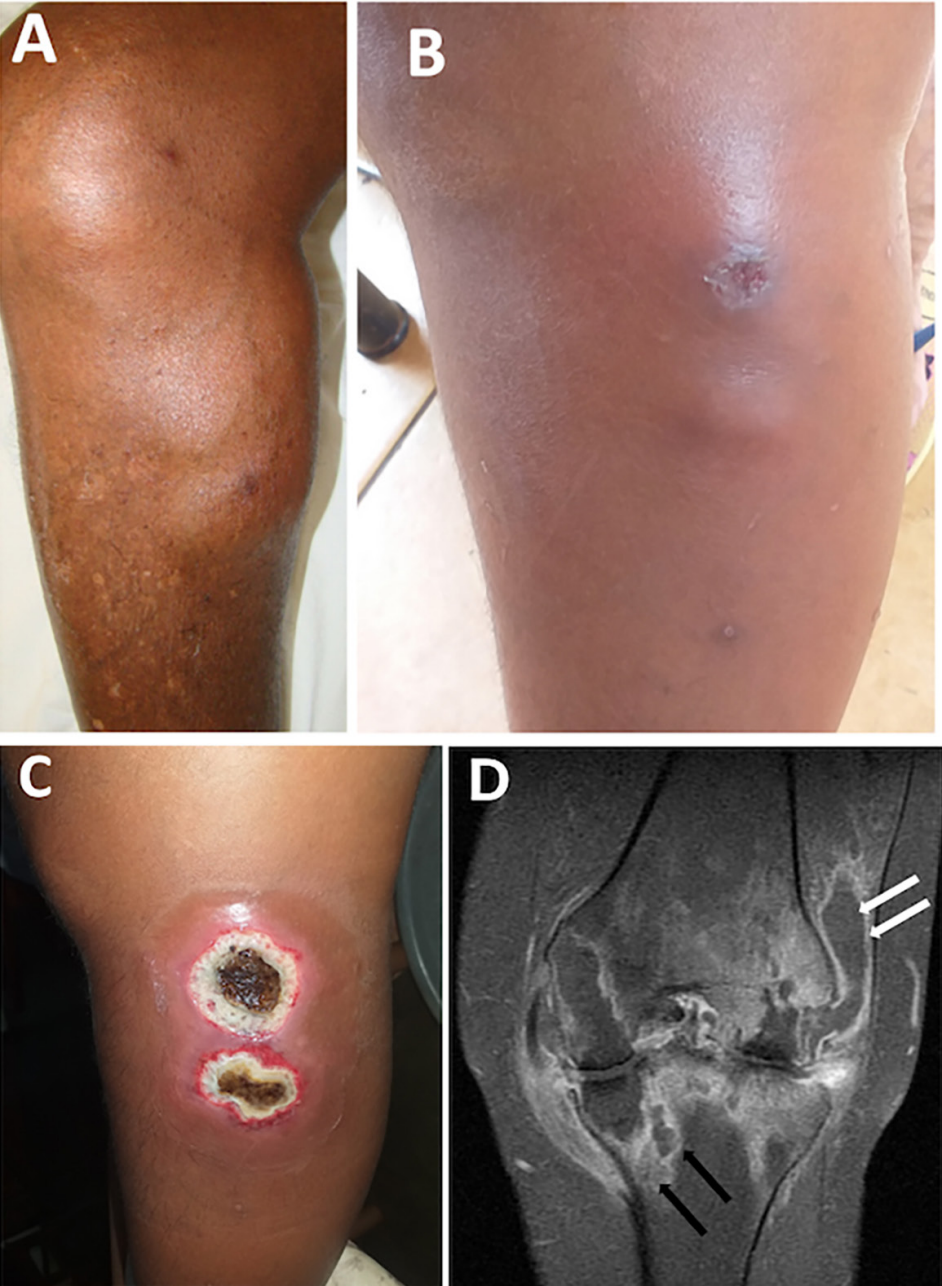
Clinical and radiological findings in patients with osteoarticular sporotrichosis: (A) A 67-year-old man with a subcutaneous abscess in the upper medial portion of the right leg (Case 4); (B, C) A 39-year-old woman with nodular and ulcerative skin lesions on the lateral aspect of the left leg, adjacent to the knee, after exposure to a sick cat, with progression of the lesions over a few weeks (Case 5); (D) Magnetic resonance imaging of the left knee (Case 5). Coronal T1-weighted image with contrast and fat saturation shows femorotibial arthropathy with tibial bone marrow edema and ring-enhancing lesions (black arrows), as well as a collection beneath the iliotibial tract extending into the joint cavity (white arrows).

A 67-year-old rural worker with a history of tuberculosis, smoking, and alcoholism developed chronic right knee pain and swelling, progressively limiting joint mobility over three years. He reported occupational exposure to soil and vegetation. Examination revealed edema, restricted extension, and a 15 cm abscess on the upper-medial leg (Figure 1A). HIV serology was negative. *Sporothrix* spp. was isolated from abscess fluid and from bone fragments obtained during surgical drainage, confirming osteoarticular sporotrichosis. He was treated with itraconazole (400 mg/day) for 30 months, resulting in partial improvement. Five years later, the knee remained enlarged with residual joint stiffness, consistent with sequelae of unifocal osteoarticular sporotrichosis. Anti-*Sporothrix* antibodies declined from 1:32 at diagnosis to 1:8 at final evaluation.

### Case 5

A 39-year-old domestic worker with a history of smoking and chronic alcohol consumption developed progressive pain, edema, and functional limitation of the left knee during a year. The condition began with an ulcerated lesion on the lateral aspect of the knee, which progressed to tissue necrosis (Figures 1B and 1C). She reported raising domestic cats at home, one of which had been ill during the same period. HIV serology was negative. Surgical debridement and bone sequestrectomy were performed, followed by placement of a transarticular external fixator. Despite these interventions, the patient continued to experience joint swelling, pain with movement, and restricted mobility. Magnetic resonance imaging revealed signs of osteomyelitis and septic arthritis (Figure 1D). *Sporothrix* spp. was isolated from bone and synovial tissue obtained during surgery, confirming unifocal osteoarticular sporotrichosis. Serologic testing showed antibody titers of 1:64 prior to treatment. Oral itraconazole (400 mg/day) was initiated, resulting in progressive improvement of joint inflammation and complete healing of the skin lesion. However, residual functional loss of the knee persisted. Follow-up serology demonstrated a decline in antibody titers from 1:64 to 1:16.

## DISCUSSION

This case series illustrates the clinical heterogeneity and diagnostic complexity of osteoarticular sporotrichosis. The five patients had chronic or recurrent arthritis, manifesting as mono- or polyarthritis of wrists, small joints of the hands, and knees. The nonspecific nature of symptoms often mimicked rheumatologic diseases, leading to diagnostic delays. Fungal osteomyelitis also commonly exists in contiguous bones, in addition to thickening of the tendon sheath, cysts, and periarticular abscess, with eventual fistulization^3^.

Most cases in this series presented primary or unifocal osteoarticular sporotrichosis, which may follow two clinical patterns. In the first, joint involvement arises concurrently to or soon after cutaneous or subcutaneous lesions, as observed in Cases 2, 4, and 5. Cases often resulting from trauma with plant material or zoonotic exposure are more readily recognized, particularly in endemic regions or during epidemic outbreaks of sporotrichosis^7^. Bone and joint lesions result from the contiguous spread of the fungal infection^3^. In the second pattern of the primary type, joint symptoms emerge in isolation, without prior skin lesions or obvious inoculation history (Cases 1 and 4). Such presentations are often misdiagnosed as rheumatologic conditions, leading to inappropriate immunosuppressive therapy that can exacerbate fungal infection^8^. An even greater challenge is the recognition of opportunistic osteoarticular sporotrichosis in patients with rheumatoid arthritis, as the clinical manifestations can be indistinguishable^9^. Case 3, in contrast, involved a patient with diabetes mellitus who presented presumed multifocal or systemic form, characterized by hematogenous dissemination of the fungus to multiple osteoarticular sites and is typically associated with immunosuppressive conditions such as HIV infection, chronic alcoholism, diabetes, malnutrition, and drug-induced immunosuppression^3^. In a study evaluating risk factors for systemic sporotrichosis in Brazil, Magalhães *et al*.^10^. identified HIV infection, alcoholism, and diabetes as significant risk factors, in which diabetes was associated explicitly with osteoarticular involvement.

Joint manifestations may also occur without direct fungal invasion, representing the immunoreactive type. In these cases, arthritis and associated findings, such as erythema nodosum or other reactive skin lesions, are thought to result from a hypersensitivity reaction to *Sporothrix* spp.^11^.

While most cases were likely related to sapronosis transmission via environmental exposure (cases 1 to 4), one case was attributed to zoonotic transmission from a sick domestic cat (case 5). This finding is relevant in Brazil, where feline-transmitted sporotrichosis has become increasingly prevalent and sporotrichosis is hyperendemic^2^.

Diagnostic confirmation was primarily achieved via fungal culture of synovial fluid, abscess material, or bone tissue. Histopathology and serological testing served as useful complementary methods. Cytological analysis of synovial fluid may lead to diagnostic uncertainty, as both polymorphonuclear and mononuclear patterns can be observed^12^. Histopathological examination often reveals mononuclear infiltrates, although fungal elements are not consistently seen in tissue sections^13^.

The search for anti-*Sporothrix* spp antibodies using methods such as ELISA and counterimmunoelectrophoresis has supported the diagnosis of patients with disseminated sporotrichosis, including those with chronic osteoarthritis, mainly when there are no skin lesions in disseminated types or when tissue sampling was unfeasible^14^. Nevertheless, serology alone is not definitive due to the possibility of false-positive and false-negative results. In this case series, declining antibody titers during follow-up correlated with a favorable treatment response. However, serological titration is not yet validated as a cure criterion and should be interpreted as a supportive diagnostic method in the clinical and mycological context.

*Sporothrix* spp., including the predominant Brazilian species of *S. brasiliensis*, is generally susceptible in vitro to itraconazole, amphotericin B, and terbinafine, whereas fluconazole and voriconazole show lower activity^15^. Cutaneous types of sporotrichosis typically respond to oral itraconazole for three to six months, but disseminated cases, such as osteoarticular involvement, often require initial treatment with amphotericin B^16^. Itraconazole at 400 mg/day is the standard regimen for osteoarticular cases, with a possible dose reduction to 200 mg/day during maintenance. Treatment should extend for 12–18 months and be discontinued only upon clinical, radiological, and laboratory resolution^17^. In this series, two patients experienced relapses (cases 2 and 3), likely due to poor adherence or absorption. Liposomal amphotericin B was used after relapse with itraconazole, showing initial response but later recurrence. Intra-articular amphotericin B was successfully employed in one refractory case in this series and may represent a valuable therapeutic option for managing relapsed or treatment-resistant osteoarticular sporotrichosis^4^. Posaconazole has been effective in itraconazole-refractory infections^18^. Combination therapy with itraconazole or saturated solution of potassium iodide plus sulfamethoxazole/trimethoprim has also been reported in osteoarticular sporotrichosis, with favorable outcomes in case reports^19,20^. Although inexpensive and widely available, this regimen lacks validation in controlled clinical studies. Surgical procedures such as synovectomy, arthrodesis, or debridement may be required in selected cases.

Given the chronic and destructive nature of the disease, sequelae with impaired joint function are common even after mycological cure. In such situations, surgical debridement represents an important adjunctive measure in infectious arthritis and osteomyelitis, facilitating disease control and supporting functional recovery^3^.

This study has several limitations. First, the small number of cases limits the generalization of the findings and prevents robust conclusions regarding therapeutic efficacy and clinical outcomes. Second, species-level identification of *Sporothrix* was not performed, as molecular diagnostic methods were unavailable during part of the study period. This limitation reduces the ability to correlate clinical features and transmission patterns with specific *Sporothrix* species. Moreover, the retrospective nature of the study may have led to incomplete clinical documentation or underreporting of relevant exposures. Despite these limitations, the detailed clinical descriptions and long-term follow-up contribute valuable information to understanding osteoarticular sporotrichosis, an underrecognized and challenging type of fungal infection.

## CONCLUSION

In endemic regions for sporotrichosis, this mycosis should be included in the differential diagnosis of chronic musculoskeletal conditions, including arthritis, osteomyelitis, synovitis, bursitis, and tenosynovitis. The definitive diagnosis relies on fungal culture, with serological and histopathological methods as complementary approaches. Treatment requires prolonged antifungal therapy, with surgical management needed in some cases, and may still be complicated by relapses and joint sequelae.

## Data Availability

The authors will not make additional data available due to ethical restrictions related to patient confidentiality.
